# Lost in care pathway: a qualitative investigation on the health system delay of extra pulmonary tuberculosis patients in Bangladesh

**DOI:** 10.1186/s12913-017-2181-8

**Published:** 2017-03-28

**Authors:** Malabika Sarker, Din Mohammad, Sukanta Paul, Rahima Akter, Shayla Islam, Goutam Biswas, Asheque Hossain, Akramul Islam

**Affiliations:** 1 James P. Grant School of Public Health, BRAC University, 68 Mohakhali, Dhaka, 1212 Bangladesh; 2 Department of Media Studies and Journalism, University of Liberal Arts Bangladesh, House 56, Road 4/A, Dhanmondi, Dhaka, 1209 Bangladesh; 3 Health Nutrition and Population Programme, BRAC, 75 Mohakhali, Dhaka, 1212 Bangladesh; 4 National Tuberculosis Control Programme, Directorate General of Health Services, Mohakhali, Dhaka, 1212 Bangladesh

**Keywords:** Extra pulmonary, Tuberculosis, EPTB, Healthcare-seeking pathway, DOTS, Bangladesh

## Abstract

**Background:**

Although extra pulmonary tuberculosis (EPTB) has long been known as a major public health concern globally, the complex healthcare-seeking pathways of EPTB patients are not widely studied. This study aims to explore the pattern of healthcare-seeking pathways of rural and urban EPTB patients registered with the BRAC TB control programme. BRAC is a Bangladesh-based non-governmental organization dedicated to alleviating poverty through empowering the poor.

**Method:**

Data were collected through 60 in-depth interviews with rural and urban EPTB patients in Bangladesh.

**Results:**

The findings reveal that the rural EPTB patients encountered a substantial diagnostic delay as compared to the urban patients. However, the difference between the average number of healthcare providers consulted by the rural verses the urban EPTB patients was not significant. This study also shows that the healthcare-seeking journey of rural and urban EPTB patients usually starts either at pharmacies or private facilities. Through exploring the detailed nature of the pathway, this study reveals the ways in which non-medical informants, mainly relatives and friends, can benefit patients.

**Conclusions:**

The private and informal healthcare providers should receive appropriate training on the diagnosis of EPTB. Such training could effectively shorten the long and complex healthcare-seeking pathways of EPTB patients.

**Electronic supplementary material:**

The online version of this article (doi:10.1186/s12913-017-2181-8) contains supplementary material, which is available to authorized users.

## Background

Extra pulmonary tuberculosis (EPTB) is a global public health problem. EPTB’s broad spectrum of clinical manifestation remains grossly underreported [1]. The most common site for tuberculosis (TB) disease is the lungs, hence the disease is known as pulmonary TB. However TB can also spread to other organs, specifically lymph nodes, abdomen, and bones and joints, including the spine [2]. Timely diagnosis and early treatment are important for an effective TB control programme. Delay in seeking care can cause patients further complications and early death [3–5]. Factors contributing to delay in diagnosis and treatment of EPTB patients can widely vary depending on population and context. A few studies have explored factors associated with EPTB and health system delay [6, 7]. The literature suggests that one of the most common health system delays occurs in the case of EPTB, in which the DOT (Directly Observed Treatment) center is not considered as the first destination for healthcare [8]. In many DOT cases, the treatment starts with an informal medical service provider [9]. A review of literature on healthcare-seeking pathway (HcSP) of TB patients reveals substantial treatment delay for pulmonary TB patients in India, China, and Bangladesh [10–12]. One study on the HcSP of EPTB patients in India concludes that accessing different medical facilities creates a significant EPTB treatment delay [13].

The prevalence of EPTB in Bangladesh is between 15 and 20% of all cases of TB patients [14] and there has been a gradual increase in prevalence in recent years [15–17]. Despite success in TB control, the diagnosis and treatment of EPTB in Bangladesh remains a challenge due to the disease’s obscure nature [18]. In addition, the gross incidence of inappropriate treatment and inaccurate referrals, mainly by rural practitioners, generally leads EPTB patients to a long and lengthy healthcare-seeking pathways [19].

In terms of health care seeking behavior, Schumann has formulated a systematic approach of studying five key stages of illness and medical care. This systematic approach is complemented by Andersen’s health care seeking behavior model [20, 21]. Andersen asserts that primary determinants are the demographic pattern of the healthcare-seeking person and the structure and organization of the healthcare system [21]. Schumann proposes that a person’s healthcare-seeking pattern is determined by his or her membership within parochial and cosmopolitan social networks [22]. However, these models were criticized for their linear and rigid nature [23]. The joint application of both models can provide comprensive information around health care seeking pathways of a patient. These pathways are determined by initiation of a symptom according to Schumann and the organization of a health care system according to Anderson.

Until now, no study has explored the HcSP of EPTB patients in Bangladesh. This paper presents a qualitative investigation of the pattern of HcSP for EPTB patients enrolled in the BRAC DOTS (Directly Observed Treatment, Short-course) programme during January 2014 to March 2014. BRAC is the largest non-governmental organization in the world dedicated to alleviating poverty by empowering the poor (www.brac.net). This study attempts to capture the non-linear paths of EPTB patients from the onset of symptoms to the process of diagnosis and treatment at the DOTS center. The next part of the paper provides an in-depth understanding of the pathways that bring about EPTB patient suffering due to extensive visitations to multiple health providers before being appropriately diagnosed and treated. This paper explores the factors that positively contribute in the delay and its pattern in order to suggest appropriate points of intervention for combating the diagnostic delay of EPTB in Bangladesh.

## Method

### Setting

A qualitative study design was best suited for the objectives based on the exploratory nature of the study. The study sought to interpret the meaning that respondents attach to their healthcare-seeking pathway and related experiences. In early 2014, six interviewers (including three authors) collected data on the healthcare-seeking pathways of EPTB patients in both rural and urban locations where BRAC operates its community based TB control programme in Bangladesh. For rural locations, coastal, hilly and plain land areas were chosen while two slums of Dhaka city were selected as urban locations. All five locations were chosen purposively.

### Research instruments and sampling procedure

In-depth interviewing was planned as the main instrument for data collection because of a specific focus on particular characteristics (healthcare-seeking pathway) of the population (EPTB patients). Data were collected through single, audio-recorded, face-to-face in-depth interviews (IDIs) with EPTB patients using semi-structured guidelines (see Additional file 1). Interviewers were trained in qualitative data collection before conducting the interviews. The duration of each interview was around 40 min. Interview domains, aimed at the study’s broader objectives, tapped into pathways undertaken by the respondents, while focusing on types of interim contact points, roles of non-medical informants, rural-urban differences, and time from onset of symptoms to diagnosis, and delay and switching between healthcare providers.

**Fig. 1:**
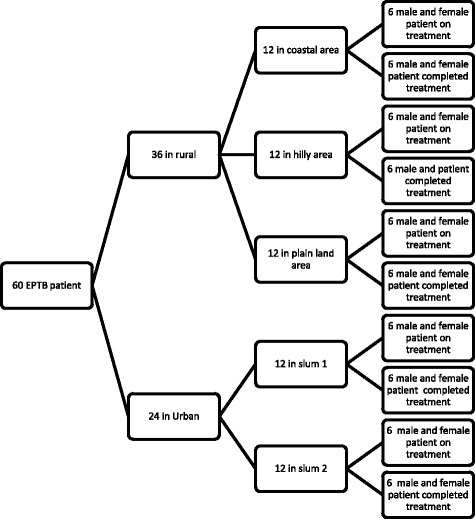
Sampling design

The sampling procedure was stratified and purposive. The aim was to conduct an analysis which would allow for investigation of variations in healthcare-seeking experience, the rationale being that any common pattern emerging from such a variation will provide powerful insight into the research question [24]. In the sampling process equal weight was given to a) locality (rural and urban), b) gender, and c) patients’ condition (under treatment and completed treatment). The respondents were selected from the register of the BRAC TB Control Programme with the assistance of BRAC staff. After conducting 36 IDIs with EPTB patients in rural areas and 24 IDIs with EPTB patients in urban areas (Fig. 1), adequate data saturation level had been reached.

### Data analysis procedure

Data were recorded using digital voice recorders and thorough field notes were taken during data collection. A qualitative research team independently transcribed the interviews verbatim in Bangla. When they experienced noise and interruptions or unclear conversation while listening to the audio recordings during transcription, field notes were checked and merged with the transcriptions to ensure data quality. The transcribed data were read, re-read, and then reviewed by all the researchers independently for familiarity with the data and to clarify any confusion. The Bangla texts were analysed using the “contrast and compare” method rooted in grounded theory [25]. The authors then discussed with other researchers and developed an initial categorization by using thematic analysis [26].

Categories and sub-categories were developed, modified and expanded on the basis of frequency and prominence of certain themes as they emerged in the data analysis process. Links between categories were progressively identified to illuminate the understanding of the research question. Analyst triangulation [24] was applied after three authors conducted the analysis. The research team comprised of researchers from Social Science or Anthropology backgrounds with prior experience collecting and analyzing qualitative data.

**Fig. 2:**
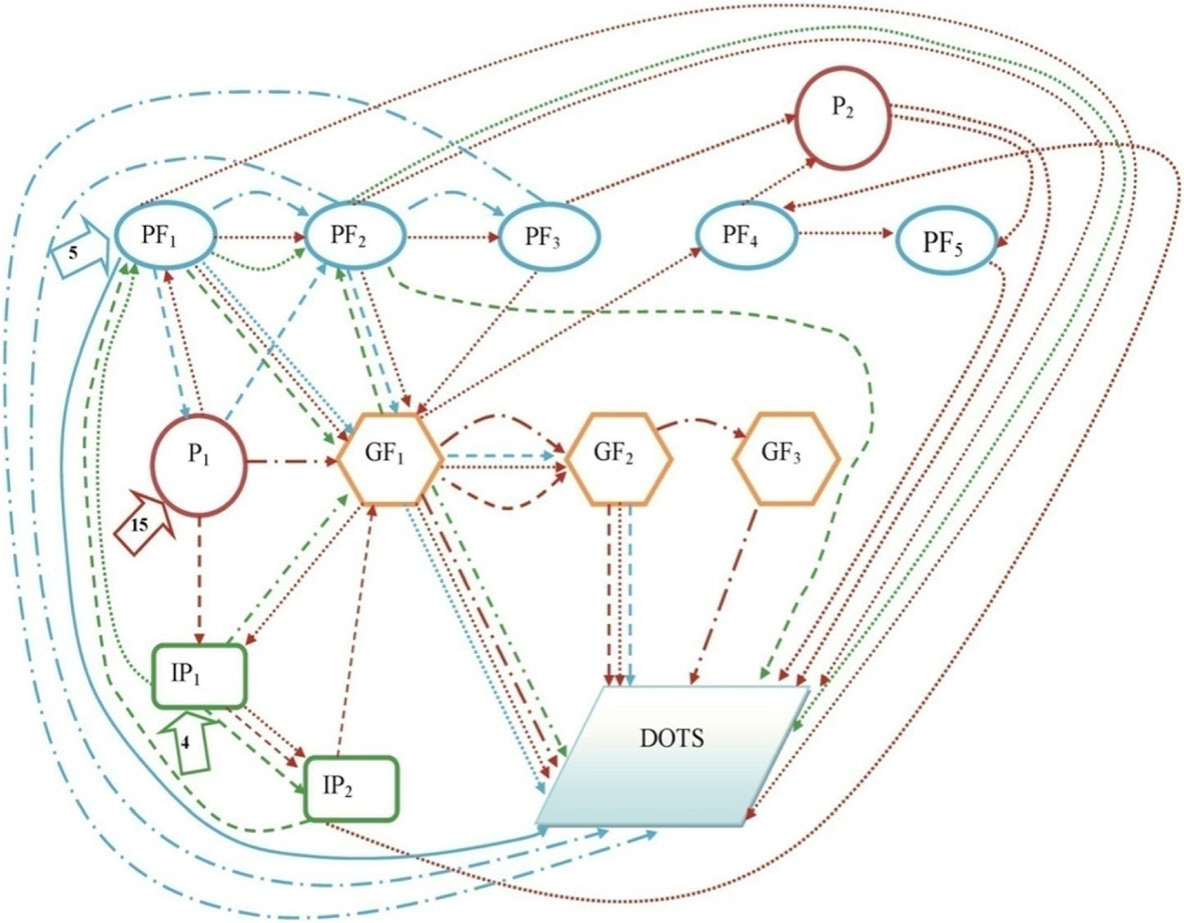
Healthcare-seeking pathways of EPTB patients in urban areas. P_1,_ P_2_ –1^st^ and 2^nd^ Pharmacy visited by the patient. PF_1,_ PF_2_, PF_3,_ PF_4,_ PF_5_ –1^st^, 2^nd^, 3^rd^, 4^th^ and 5^th^ private facility visited by the patient. IP_1,_ IP_2_ –1^st^ and 2^nd^ informal provider visited by the patient. GF_1,_ GF_2_, GF_3_ –1^st^, 2^nd^ and 3^rd^ Govt. facility visited by the patient. The number mentioned inside the arrows () describe the number of patients visiting 1^st^ contact

**Fig. 3:**
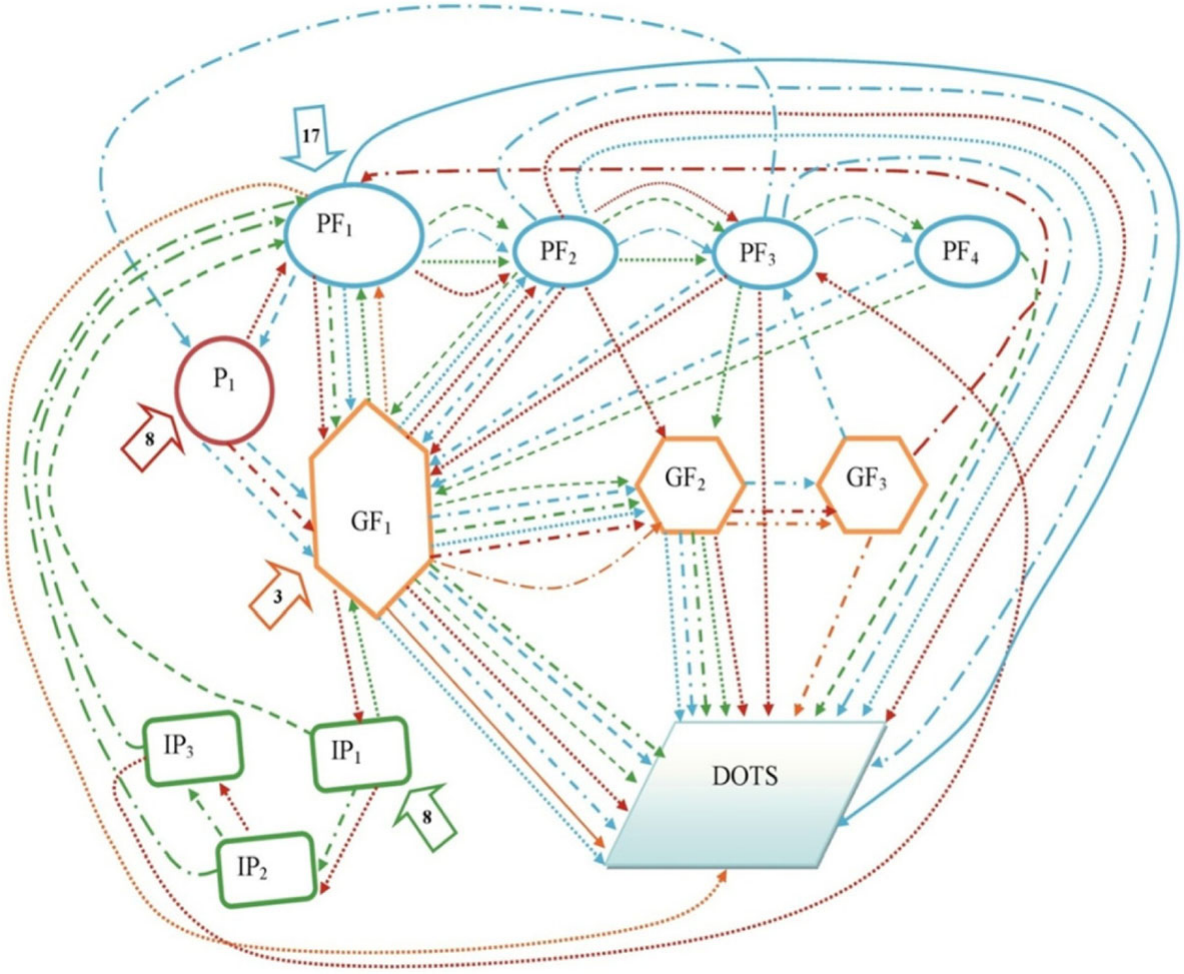
Healthcare-seeking pathways of EPTB patients in rural areas. P1–1^st^ Pharmacy visited by the patient. PF1, PF2, PF3, PF4–1^st^, 2^nd^, 3^rd^ and 4^th^ private facility visited by the patient. IP1, IP2, IP3–1^st^, 2^nd^ and 3^rd^ informal provider visited by the patient. GF1, GF2, GF3–1^st^, 2^nd^ and 3^rd^ Govt. facility visited by the patient. The number mentioned inside the arrows () describe the number of patients visiting 1^st^ contact

Findings presented in Tables 1 and 2 and Figs. 2 and 3 were obtained through re-reading each and every transcripts. Three co-authors sat together and identified the pathways and referrals for 60 in-depth interviews in both urban and rural areas. After that they placed the data in a matrix using Microsoft word. Later, the data was grouped, added and counted for presentation in the format of Tables 1 and 2, Figs. 2 and 3.

**Table 1:**
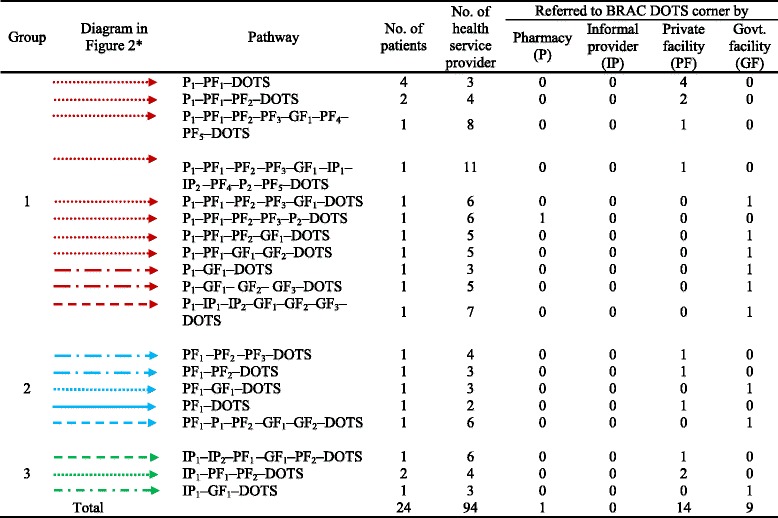
Pathways and referrals of the extra pulmonary TB patients in urban areas

*The shapes and colors of the arrows are differentiated based on the *second* contact point of the patient

**Table 2:**
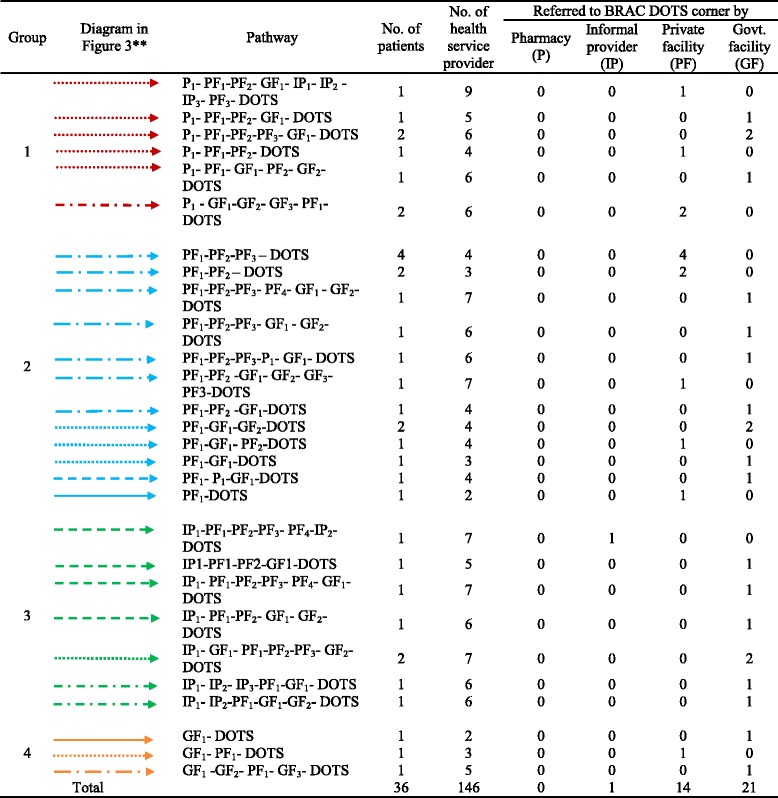
Pathways and referral of the extra pulmonary TB patients in rural areas

**The shapes and colors of the arrows are differentiated based on the *second* contact point of the patient

To ensure trustworthiness and rigor of the data various strategies were adopted. Two of the co-authors closely supervised and observed the interviews randomly during field work. They have also participated in the data collection process. Peer debriefing sessions were held everyday, in the evenings, during the data collection period to induce further ideas, probes, and to modify the guidelines [27, 28].

Regarding reflexivity, interviewers felt emotionally attached to some EPTB patients who had undergone significant suffering during illness periods. The research team was male except for one female researcher. As a result, female EPTB patients may have felt uncomfortable while being interviewed by a researcher of opposite gender.

Ethical approval was obtained from James P. Grant School of Public Health (JPGSPH), BRAC University. In addition, formal consent was taken from the BRAC TB control programme by providing a letter of authorization, including the study details prior to data collection. As JPGSPH is an umbrella organization of BRAC, no administrative barrier was experienced in this regard. BRAC local level staff contacted TB patients and informed them about the study and their voluntary participation. BRAC staff only provided the information of those who agreed to participate in the research. Verbal consent was taken from each respondent after clarifying the voluntary nature of their participation in the study and the right to leave the study at any time. After taking consent, the interviewers checked the ‘yes’ box on the paper. The interviewers also informed participants about the confidentiality policy. Furthermore, the study guidelines were anonymous and the interviews were conducted in a private setting to provide the respondents with their right to privacy and breathing space for sensitive questions. All participants agreed to be interviewed. The participantes were recruited first through BRAC staff working at DOT centers and then through voluntary participation. Interviews were carried out at a time and place convenient for the participants once their trust was gained.

## Results

The findings presented below are organized in four themes. The first theme draws a detailed graphical presentation of the healthcare-seeking pathways for urban and rural patients separately. The following themes briefly present the types of interim medical contacts consulted, the helping role of non-medical informants in finding the correct destination, and the perceived and reported reasons for switching between health providers. Verbatim quotes were obtained and recorded in Bangla, and then translated into English by the authors.

### Healthcare-seeking pathways

We have drawn out the healthcare-seeking pathways through showing the medical contact points visited by each respondent. These contact points are divided into four categories – pharmacies, private facilities, informal providers, and government facilities. DOTS is the fifth medical contact point where the journey of EPTB patients ends. We divided both the urban and rural pathways into different clusters by using different colors for each of the initial contact points. Consequentially, we used different types of arrows to signify different paths, mainly on the basis of the type of second contact (Tables 1 and 2). Similar legends were used for urban and rural pathways (Figs. 2 and 3).

#### Pathways followed by urban patients

Pharmacies or medicine shops were reported as the first contact point by most of the urban respondents. Based on the extended conversation and clues given in the interviews, perceived reasons behind choosing pharmacies as the initial medical destination were:

- Accessibility (as pharmacies are located mostly in the neighborhood)
- Acceptability (as drug-sellers are perceived as knowledgeable about disease and treatments)
- Affordability (as no consultancy fee is required at pharmacies)

As mentioned by one of the respondents,

*“We are poor. Our income is very low. When we fell sick, we always visit the place where the medical cost is lower… the pharmacy guy is very helpful. His medicine works better.”* (An urban male EPTB patient under treatment)

While 15 urban respondents who consulted pharmacies initially, four respondents chose informal providers and five chose private facilities as their initial contact points. No one initially sought treatment from government facilities. BRAC DOTS centers were not the first contact point for any of the respondents in both urban and rural areas. None of the informal providers and only two of the pharmacies referred patients directly to the DOTS centre (Fig. 2).

The longest pathway we observed was of an urban female who visited 11 health service providers before being referred to the DOTS centre.

Fourteen out of 24 patients were referred to a BRAC DOTS centre by private facilities, whereas nine patients were referred to BRAC DOTS centers by government facilities (Table 1).

#### Pathways followed by rural patients

The majority of respondents from rural areas sought graduate doctors in private facilities as their first medical contacts. Such facilities are commonly located in nearby towns. Patients accessed a qualified doctor in hope of fast healing. As mentioned by a rural respondent,

*“I think, nowadays, if someone experiences any health problem such as fever, bodily pain, skin disease or anything then he/she should first go to the MBBS doctor (qualified doctor). We always go to Dr. X and his medicine works fine for us. We recover fast by following his advice and medicine…”.* (A rural male EPTB patient under treatment)

Other than MBBS (Bachelor of Medicine, Bachelor of Surgery) doctors in private facilities, pharmacies were mentioned as the initial medical contact point by eight respondents. Eight other respondents initially consulted informal providers. Three out of 36 patients started seeking treatments from the government facilities. No one accessed a BRAC DOTS center during their initial stage of illness. Patients were mostly referred to DOTS centres by the government and private facilities. One out of the 16 respondents seeking initial consultation from pharmacies and informal providers was referred to DOTS (Fig. 3).

### Types of interim contact points

The respondents visited several health facilities in various orders and none of these orders appeared to have a combination benefitical for effectively minimizing the time of the pathway. Although we divided the medical contact points visited by EPTB patients into four types, the contact points sometimes do not mean exactly the same thing in rural and urban settings. For example, “informal provider” signifies a number of health contact points in rural areas, such as village doctors, traditional herbal practitioners, homeopaths, *shamans* or spiritual healers. However, in urban areas, the respondents did not meet spiritual healers or rarely met herbal practitioners. For the urban respondents, unqualified medical practitioners were labeled as ‘informal providers’. Private facilities may mean private chambers or private clinics in both settings, although rural respondents mostly went to private chambers and urban respondents to private clinics. Pharmacies had the same meaning in both settings, although the size of the pharmacy, collection of medicine, and the education of the druggist/chemist considerably varied between rural and urban areas.

In most cases, the respondents followed a path which started from the non-formal medical contact points to the formal health facilities. However, in a few cases, it was the other way around where participants gave up visiting formal health facilities and returned to the informal health providers. One participant stated,

*“Initially I took Bonaji oushodh (herbal medicines) for two weeks. Then I have taken treatment from some boro daktar (specialist doctor) at the nearby hospitals. Everyone has given me a lot of medicines but my physical condition was getting worse day by day…after that, without seeing any improvement, I went to a local healer and started taking Bonaji medicines again…”.* (A treatment completed rural male EPTB patient)

This is what made healthcare-seeking pathways unusually long and full of suffering. The next theme presents the reasons for the travel between health facilities.

### Perceived reasons for diagnostic delay and switching between health providers

Diagnostic delay, however short or long, was reported by all the respondents in both rural and urban areas. The shortest duration between the appearance of symptoms and diagnosis was one month, which happened in 16 out of 60 cases. The longest duration from symptoms to diagnosis was 48 months for one rural female respondent. The average time spent for diagnosing urban EPTB patients was 13 and 28 weeks for rural EPTB patients. The average number of service providers that the urban and rural patient visited before being referred to a DOTS center was very similar, 2.91 and 3.05 respectively.

What made the healthcare pathways so long? When asked, 22 of 60 respondents stated that they switched between different types of service providers as the illness persisted. Therefore, according to them, none of the services sought could bring conclusion. Moreover, in most cases, previous healthcare experiences did not contribute positively in the next healthcare experience because the structure of the healthcare was entirely different (e.g. from herbal to private formal, etc.). Some informants mentioned their dissatisfaction with village doctors, which led them to visit MBBS doctors in hospitals. But after a long period, generally the village doctors preferred to continue the treatment rather than refer the patient to a health facility or a trained physician. One of the respondents expressed,

*“Around six months, I took medicines from a polli doctor (village doctor) for fever, cold-cough and pain in my leg. After two or three months I asked him, do I have any other disease doctor? Do I have to consult with any other doctor? By giving some medicines, he said, take these medicines, you will be fine…but nothing has changed…he held me up for six months”.* (A rural male EPTB patient under treatment)

A few respondents mentioned that financial constraints were a main reason for switching between service providers, since they could not afford the cost of formal health facilities. Moving from formal medical providers to informal providers resulted in long and complex pathways, as mentioned by one of the respondents:

*“I was exhausted after taking so many medicines one after one. At first I visited a village doctor and took medicines from the pharmacy. After that I consulted two MBBS doctors. But no drugs worked for me. Then my aunt suggested me to go to Comilla (a district) where I was treated with oil, water and herbal medicines by a hakeem (traditional healer).”* (A treatment completed urban male EPTB patient)

Several respondents mentioned that the misreading of their symptoms by health providers was a reason for delay. This not only happened with informal providers, but in some cases with MBBS doctors as well. As mentioned by one respondent,

*“I had to take the decision by myself because my husband was outside the country. In December 2012 I went to the pharmacy with fever and stomach ache and they provided me some medicine. But it did not work well and I decided to visit an MBBS doctor. Instead of reading the symptoms accurately, he advised me treatment for gastric ulcer. Thus, spending a long time, consulting with many doctors instead of my health being improved I became more sick. Then I thought I should not take any medicine. Finally, in September 2013, after nine months of sufferings, I was diagnosed as a TB patient.”* (A rural female EPTB patient under treatment)

In addition, lack of knowledge, lack of awareness and education of family members, commercial attitudes of doctors, and personal fear were also reported as important reasons for diagnostic delay by respondents in both rural and urban areas.

### Role of non-medical informants

The eye-opening role of family members, neighbours, friends and relatives was often mentioned by respondents as instrumental in the process of gaining a diagnosis and treatment. Respondents were often advised by non-medical friends and family members. Most of this advice was on how to access qualified doctors or BRAC clinics, except in one case where a relative advised a respondent to go to an informal provider. As mentioned by a respondent,

*“I was moving from one doctor to another, but getting no cure. My next door aunt was a real witness of my sufferings. One day she advised me to go to the BRAC office. But I was confused. After visiting some other pharmacies, I went to the X Hospital (a renowned private hospital in Dhaka). After conducting a TB test, they diagnosed my TB. Then, I was referred to BRAC.”* (An urban female EPTB patient under treatment)

However, the role of non-medical friends and family members was not only limited advising on where to access care. Non-medical friends and family members also played an important role in providing financial support for treatment.

## Discussion

In order to understand the healthcare-seeking pathway of EPTB patients, this study examined the experience of EPTB patients in both urban and rural areas in Bangladesh. The findings presented the range and complexity of the pathways undertaken, which was caused by the tendency to switch between providers, the dealy in diagnosis and treatment, and financial challenges.

The average time spent in diagnosing EPTB was 13 weeks for urban patients and 28 weeks for rural patients. This finding is consistent with similar studies conducted in urban India and Norway where the median delay in healthcare-seeking for an EPTB patient was estimated to be 12 and 11.5 weeks, respectively [13, 29]. However, the average number of service providers visited before accessing a DOTS center was 2.91 in urban and 3.05 in rural areas. Here, the difference between the number of service providers visited by urban and rural patients is much smaller when i compared to the difference between the period of delay observed in rural verses urban areas. This implies that service providers tend to hold a patient for an unusually long time even if the case that diagnosis was not successful. Admittedly, the obscure nature of EPTB makes it equally difficult to be diagnosed by general medical practitioners irrespective of rural or urban affiliations, unless and until medical practitioners have specific training and information on this disease. At the same time, it should also be noted that financial hardship and patient tendency to switch between different medical services (allopath to homeopath or herbal) contributed significantly in the process of delay. The difference between average delay for EPTB patients in rural and urban areas was significant, which signifies not only the difference between urban and rural healthcare infrastructure, but also the promptness of urban patients in taking decisions compared to their rural counterparts.

In urban areas, pharmacies were mentioned as the first contact point for most of the EPTB patients. This finding is consistent with another study conducted in Delhi, where drugsellers were the first point of contact and source of clinical advice for two-thirds of the patients [10]. The pathways also showed that almost none of the drug sellers and informal providers referred any EPTB patients straight to a DOTS centre. The hard-to-detect nature of EPTB could be one of the main reasons behind this. But it should also be noted that the awareness of the informal providers has not improved even in the case of pulmonary TB, despite continuous efforts being undertaken through the NTP (National Tuberculosis Programe) training program under public-private mix (PPM) [30]. However, in rural areas, the majority of the respondents visited qualified practitioners in private facilities. Similar results were observed elsewhere, which reconfirm the importance of sensitizing private practitioners to EPTB in rural areas [13]. At the same time, more careful strategies should be worked out to ensure the efficiency of informal providers in urban areas, so that they can be more specific about the nature of EPTB.

As we can observe, most of the rural EPTB patients were referred to DOTS services by the government facilities, whereas most of the urban patients were referred by the private facilities. Studies elsewhere also confirmed that referrals from the government facilities were higher than the private facilities which indicates the programme’s success in the government sector [13, 31]. At the same time, as is evident by the present study, the non-government health infrastructure in rural areas is still incompetent in dealing with cases of EPTB, whereas in urban areas the situation has significantly improved. However, appropriate strategies are needed to involve qualified practitioners as key stakeholders in public-private mix strategy and provide necessary information through an appropriate health communication measure.

Another finding of this study was that family members and friends play a somewhat crucial role as financers and advisors in the process of diagnosis and treatment of EPTB patients.. In many cases, these non-medical informants were able to correctly advise the EPTB patients about contacting the DOTS centre and thereby contributed in the process of stopping further expansion of pathways. The EPTB health communication measures should seek out a strategy which would involve these people as important stakeholders. Further studies can be carried out to explore the information-base of these people as well as who these people most commonly are, so that it can be systematically utilized in the programme design.

This study has a few limitations. Firstly, no pattern was observed among the EPTB patients based on their location, sex, and patients’ condition. Location is an important factor for EPTB patients which can significantly influence their healthcare-seeking pathways. Although we tried to make a representative sample by covering a wide variety of rural field locations, there might be individuals not covered in this study who have significant trouble accessing medical services.. This study may have recall bias in which the number of health facilities visited might be under/over-reported.

## Conclusion

The pathways followed by EPTB patients explored in this study provide valuable insight into the role of different types of healthcare providers in the diagnosis process, the types and reasons behind delays in the diagnosis, and the role of non-medical persons in this process. It is also evident that informal and private healthcare providers in rural areas should receive appropriate training on the diagnosis of EPTB, which could shorten the healthcare pathway of EPTB patients significantly, thus improving the quality of life of affected individuals.

## Additional file

Additional file 1: In-depth interview guideline. Guideline used to explore the healthcare-seeking pathway of patients with extra pulmonary tuberculosis. (DOC 47 kb)

## Abbreviations

DOT: Directly Observed Treatment
DOTS: Directly Observed Treatment, Short Course
EPTB: Extra pulmonary tuberculosis
HcSP: Healthcare seeking pathway
IDI: In-depth interview
JPGSPH: James P. Grant School of Public Health
MBBS: Bachelor of Medicine, Bachelor of Surgery
NTP: National tuberculosis programme
PPM: Public private mix
TB: Tuberculosis
