# Supplementary material for: Lost in care pathway: a qualitative investigation on the health system delay of extra pulmonary tuberculosis patients in Bangladesh
Source: BMC Health Serv Res. 2017 Mar 28;17:240. doi: 10.1186/s12913-017-2181-8 (PMC5370471; doi:10.1186/s12913-017-2181-8)
Supplement: Additional file 1: — In-depth interview guideline. Guideline used to explore the healthcare-seeking pathway of patients with extra pulmonary tuberculosis. (DOC 47 kb) [file 12913_2017_2181_MOESM1_ESM.doc]

**Additional file: In-depth interview guideline**

| Patients’ condition: | Name of the district and sub-district: |
| --- | --- |
| Duration of treatment: | Start of treatment (day): |
| Interviewer Name: | Marital Status: |
| Date of Interview: | Level of Education: |
| Occupation: | TR Number: |
| Sex: | Age: |
| Time interview Started: | Time interview ended: |

*Good morning/afternoon. My name is ______________ and this is _______________ (if 2 people present). Thank you for agreeing to speak with us. We are here to learn about the healthcare-seeking pathway of patients with extra pulmonary tuberculosis and to know the reasons for delay in treatment seeking. We are speaking with EPTB patients. Your responses will help us in our learning. If you wish, any personal or sensitive information that you choose to share with us will be kept in confidence. The interview should take no more than 45 minutes, and you should feel free to interrupt us at any time.*

*Do you have any questions? If not, we will now begin.*

*[Note: It is mendatory to take permission if you want to use voice recorder.]*

**General information**

1. How many people do you have in your family? ***Probe:*** Whom do you live with?
2. From where do you take treatment for general illness?
3. Do you know what kind of TB you have (got)? ***Probe:*** from whom, how?
4. What were the sign and symptoms? ***Probe:*** When (day/month) did you realize that you have those sign and symptoms?

**Reasons for delay in treatment seeking**

1. How many days after the appearance of symptoms you have sought treatment? ***Probe:*** How long you had to wait before treatment?
2. What were the reasons for such delay? ***Probe:*** Try to explore whether it was for issues related with ignorance, lack of knowledge etc.

**Health-care seeking pathway**

1. Where did you go for seeking treatment at first? ***Probe:*** Try to find out the initial contact points without giving any hints (pharmacists, village doctor, drug seller, traditional healer, private practitioner, DOTs corner, BRAC peripheral lab etc.).
2. Would you please describe the pathways you have taken for seeking EPTB treatment? ***Probe:*** Tell the patient to narrate his/her story (*chronologically)* following the steps given below:

- When did the event occur? (Ask specifically about the time)
- Ask about the sign and symptoms.
- Went to which service provider?
- What kind of treatment they have given?
- Whether the patient have switched to another service provider or not? If yes, then what were the reasons behind going to another service provider?
- Ask about the health care expenditure occurred. How did you manage to bear the cost?

1. Do you take or have taken treatment from any other service provider during the treatment period? ***Probe:*** From whom you are currently taking or have taken treatment? Why?
2. Do you face or did you have to face any challenges while taking treatment? ***Probe:*** Please explain.

# Thank You
